# Who is to blame? Perspectives of caregivers on barriers to accessing healthcare for the under-fives in Butere District, Western Kenya

**DOI:** 10.1186/1471-2458-11-272

**Published:** 2011-05-03

**Authors:** Antony S Opwora, Ahmed MR Laving, Lambert O Nyabola, Joyce M Olenja

**Affiliations:** 1 Kenya Medical Research Institute/Wellcome Trust Research Programme, P.O. Box 43640-00100, Nairobi, Kenya; 2 University of Nairobi, Department of Paediatrics and Child Health, P.O. Box 19676-00202, Nairobi, Kenya; 3 University of Nairobi, Department of Community Health, P.O. Box 19676-00202, Nairobi, Kenya

## Abstract

**Background:**

Kenya, like many developing nations, continues to experience high childhood mortality in spite of the many efforts put in place by governments and international bodies to curb it. This study sought to investigate the barriers to accessing healthcare services for children aged less than five years in Butere District, a rural district experiencing high rates of mortality and morbidity despite having relatively better conditions for child survival.

**Methods:**

Exit interviews were conducted among caregivers seeking healthcare for their children in mid 2007 in all the 6 public health facilities. Additionally, views from caregivers in the community, health workers and district health managers were sought through focus group discussions (FGDs) and key informant interviews (KIs).

**Results:**

Three hundred and ninety-seven respondents were surveyed in exit interviews while 45 respondents participated in FGDs and KIs. Some practices by caregivers including early onset of child bearing, early supplementation, and utilization of traditional healers were thought to increase the risk of mortality and morbidity, although reported rates of mosquito net utilization and immunization coverage were high. The healthcare system posed barriers to access of healthcare for the under fives, through long waiting time, lack of drugs and poor services, incompetence and perceived poor attitudes of the health workers. FGDs also revealed wide-spread concerns and misconceptions about health care among the caregivers.

**Conclusion:**

Caregivers' actions were thought to influence children's progression to illness or health while the healthcare delivery system posed recurrent barriers to the accessing of healthcare for the under-fives. Actions on both fronts are necessary to reduce childhood mortality.

## Background

### Introduction

About 20 million children die every year, often from preventable causes such as acute respiratory tract infections, diarrhoea, measles, malaria and malnutrition [1,2]. Despite decades of impressive gains in the reduction of childhood mortality, many African nations have started to experience a reversal of these gains [1], sending fears among health experts that the fourth Millennium Development Goal (MDG) which aims to reduce childhood mortality by two-thirds by the year 2015 [3], may not be realized. Kenya has actually seen a reduction in the under five child mortality from 115 per 1000 live births in 2003 [4], to 74 per 1000 in 2008/9 [5]. Yet mortality remains well above the target set in the fourth MDG of about 30 per 1000 by 2015.

Numerous efforts have been put in place to reduce childhood mortality including the traditional vertical, disease-specific programs which have claimed a number of successes for example, the widespread acceptance and use of oral rehydration therapy in treating diarrhoeal diseases [6] and the increase in immunization coverage in several countries. The biggest challenge however has been with the burden they place on existing health systems and perhaps, the worst of all concerns has been the fragmented management of a child with multiple infections or health conditions [7,8].

The Integrated Management of Childhood Illnesses (IMCI) was developed and promoted by World Health Organization (WHO) and United Nations Children Fund (UNICEF) starting in the 1990's to try and mitigate some of these concerns. However, coverage is still very low, a situation attributed to lack of financial and human resources, and poor working conditions with high turnover of health workers [9] in many of the developing nations. The success of IMCI strategy in reducing childhood mortality in Kenya remains to be realized [10]. Just as in several other studies [11-13], a report evaluating the implementation and effects of IMCI recommended free access to health as a major booster to freeing barriers to health [14]. On the other hand, socio-cultural barriers to accessing health care for children through perceptions of poor quality services [15] may be playing even a bigger role. Although the health facilities may be accessible in terms of geographical and economic aspects, the services being offered might not be acceptable to caregivers of children, thus serving as a barrier.

Most of the available evidence on access barriers has been documented in hospital settings with a bias on assessing infrastructure, drugs and other medical supplies and gathering views of health personnel and managers about problems of childhood mortality. New evidence is therefore needed from caregivers who are effectively barred from accessing healthcare and also from those who experience the shortcomings of the health system.

Healthcare, as used in this study encompassed the care and provision of services that help individuals achieve an optimal state of well-being, in any setting or stage in the human life cycle [16], to include the promotive, preventive and curative services. This includes the care provided both at home and at health facilities and involves everything that caregivers and health workers do for children to promote their well-being. A broad definition of access to healthcare, which draws on the works of Obrist *et al *[17] has therefore been used. In this study, we assessed some of the barriers to accessing healthcare both in terms of care (not) provided by caregivers and their social contexts and within the health delivery system. The main objective was to determine the perceptions and practices of caregivers concerning childhood illnesses; and identify the most important barriers to accessing health care in Butere District.

## Methods

### Study Setting and Population

Butere District was carved out of the larger Butere-Mumias District, which was previously part of Kakamega District in Western Kenya. The district is typically poor and rural with very high morbidity and mortality indicators. Its location in a high potential agricultural zone with conducive climatic conditions for productive livelihood has not helped in alleviating the poverty levels or improving the health of its inhabitants.

At the time of the study, Butere had a population of 111,637 people with public health facilities comprising of one district and one sub-district hospitals, 2 health centres and 2 dispensaries [18]. Informal health care providers consisting of herbalists, traditional medicine-men, hawkers, traditional birth attendants, medical practitioners, and drug shops, many of whom were unlicensed, were also providing care. There was also a heavy presence of licensed private clinics operated by clinical officers and different cadres of nurses. The study focused on caregivers of children aged less than five years. The under-fives were chosen because they form part of the population that is most vulnerable to underlying conditions which determine the general health outlook of the population.

Being a relatively new district, data on health indicators were not available however, according to the KDHS 2003, Western Province within which the district lies, had an under-five mortality rate of 144 per 1000 live births while the vaccination rate was 50% [4], a whole ten percentage points below the national average. The KDHS 2008/9 report shows that under-five mortality has dropped by 15.9% to 121 per 1000 live births in the province, which is the second highest rate in the country (after Nyanza Province) and way above the national average of 74 per 1000 live births [5]. The same report also shows marked improvements in the vaccination coverage which, according to the report stands at 73%.

### Study Procedures

This was a descriptive cross-sectional study of caregivers of children aged below five years in Butere District. Quantitative data were collected between May and August 2007 among the caregivers attending Mother & Child Health (MCH) clinics at all the six public health facilities within the district. Each facility was allocated a proportionate number of caregivers depending on the average outpatient attendance within the month preceding the study. We aimed for an overall sample size of 385, derived from World Health Organization's formula for comparing proportions [19]. Systematic sampling was used to select caregivers who satisfied the inclusion criteria for exit interviews. During health talk sessions in the mornings, the strategy for randomly selecting the allocated number of respondents for the facility for that day was decided. For example, if the facility was allocated 10 caregivers for interview on that particular day and 50 attended the health talk morning sessions, then every fifth (5^th^) caregiver leaving the facility would be approached for interview. Only clients seeking outpatient preventive, promotive and curative services in the MCH/Child welfare clinics, who consented, were interviewed. Caregivers attending the health facility later in the day replaced selected caregivers who were excluded in the morning. Quantitative data were collected on socio-demographic characteristics of caregivers in addition to information about their practices and experiences while seeking care for their sick children. Further, we enquired about their recommendations on how to improve access to health care for children aged below five years.

Qualitative data were collected concurrently with quantitative data through Focus Group Discussions (FGD) with community members from different divisions of the district. Four FGDs were held; one in each division, to capture a diverse range of opinions from the general community. Participants were recruited through community gate keepers including church leaders (n = 2) and women's group leaders (n = 2), who invited mothers with children aged 5 years and below for a general discussion about their health. Discussions were conducted in settings within the community and at times agreed in advance with participants to ensure that participants felt as comfortable and free as possible. Each FGD consisted of between 6 and 12 caregivers with two trained enumerators to lead the discussions and to take notes. The key aspects addressed by FGDs included caregivers' perceptions of the key health problems affecting their children, their practices regarding health care for the children, experiences while seeking health care and what changes they thought were important to improve access to health care.

In addition to the FGDs described above, key informant interviews were also conducted with a district level health manager, referred to as the District Medical Officer of Health (DMOH), nurses and clinical officers working directly with the children and a traditional healer to get their perspectives of what they considered to be important caregiver and health system barriers to healthcare for the under fives, and how these barriers might be overcome.

This study was approved by the Kenyatta National Hospital/University of Nairobi Ethics Review Committee and the Ministry of Education, Science and Technology. Each interviewee received full information about the study and was asked if he/she was willing to participate before the interview began. For FGDs and individual interviews, informed consent processes included seeking permission to tape-record the proceedings. Consent was written for individual interviews and verbal for FGDs.

### Data Analysis

Quantitative data were entered into a computer using Epi-Info database and analyzed using the Statistical Package for Social Sciences version 14.0 (SPSS^©^, 1989-2005). During the FGDs and key informant interviews, notes were taken and - where possible - discussions were taped-recorded. Notes and recordings were later transcribed and where necessary translated into English. Manual analysis of the discussions was done by reading through the transcripts and extracting similar parts of the transcripts into broad themes relevant to the study objectives. Themes were identified both inductively and deductively. Initial themes were based on the interview guide questions, but new themes were identified over the course of reviewing the data. Triangulation of data was used in the analytical process. Qualitative results are presented alongside the quantitative data wherever possible.

## Results

### Socio-demographic Profile of Respondents

A total of 397 respondents were interviewed with the greatest proportion being at the Butere District Hospital (25.7%) followed by Shisaba (24.7%) and Shimukoko (16.9%) dispensaries. Shikunga and Shiraha health centres had 11.8% and 11.6% respondents, respectively. The Manyala SDH had the least number of respondents (9.3%). Forty respondents participated in four focus group discussions while 5 key informants were interviewed at various locations.

Table 1 shows the socio-demographic characteristics of exit interviewees. Out of the 397 respondents, the majority (97.7%) comprised biological mothers, whereas only 2.3% (9) were males, comprising of 8 biological fathers and 1 grandfather. A majority (61.2%) of the caregivers fell within the 20-29 years age-group. At least 13 (3.3%) respondents were aged below 18 years. Twelve (3%) respondents who did not know their age were excluded from further analyses requiring this variable.

**Table 1:** Socio-demographic Data of the Respondents

Characteristic of Respondents	Percentage
**Gender (n = 397)**	
Female	97.7
Male	2.3

**Age (n = 397)**	
Below 20	9.3
20 - 29	61.2
30 - 39	22.2
40 - 49	4.0
50 & Above	0.3
Don't Know Age	3.0

**Relation with the Child (n = 397)**	
Mother	95.7
Father	2.0
Grandparent	2.3

**Marital Status (n = 396)**	
Married, Monogamous	83.1
Married, Polygamous	8.3
Single Parent Divorced/separated/widowed	7.3 1.3

**Educational Attainment (n = 397)**	
None	5.8
Primary Incomplete	48.1
Primary Complete	32.2
Secondary	11.1
Tertiary	2.8

**Employment Status (n = 392)**	
Unemployed	33.7
Trader	20.2
Peasant Farmer	24.2
Self Employed	15.8
Formally Employed	5.6

**Level of Income (n = 397)**	
Less than Ksh 5,000	69.6
Ksh 5,000 and above	30.4

**Figure 1:**
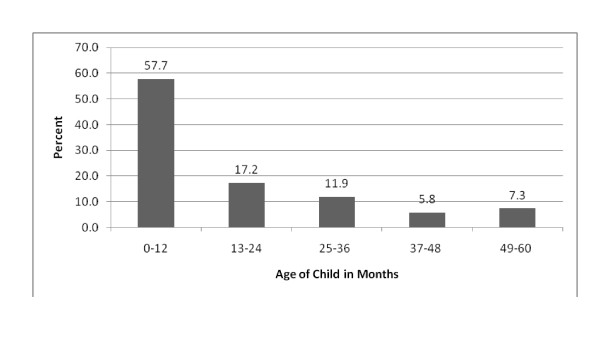
**Age Distribution of Caregivers' Children**.

Figure 1 presents the age distribution of the caregivers' children. The mean age of the children was 16.6 months (Standard Deviation (SD): 15.7) with median age of 10 months (range 1 - 60). Slightly over a half (53.5%) of the children with the caregivers were females giving a male to female ratio of 1:1.15.

### Disease Profile of Study Children and Caregivers' Perceptions and Practices Regarding their Children's Health and Illnesses

Thirty-four percent of the children were seeking routine immunization services on the interview day. Among the 256 ill children, 30.7% were reported to be suffering/have suffered in the last two weeks prior to the survey, from malaria or acute febrile illness; 17.8% from respiratory tract infection; 8.8% from a skin disorder; and 8.5% from diarrhoeal diseases. The caregivers were then asked to grade the seriousness of their children's illnesses. It was beyond the scope of this study to confirm either the diagnosis or the severity of the illnesses as judged by caregivers. Almost a half (49%) of the respondents said that their children's illnesses were severe while 38% said the illnesses were not severe. Only 13% thought the illnesses were very severe. Bivariate analysis showed that none of the maternal characteristics (age, parity, marital status, income, education and occupation), nor the child's age and gender, were associated with the caregivers' perception of the child's illness being severe or very severe. Only children with complaints other than malaria/febrile illness were more likely to be perceived as being severely or very severely ill (68.4% versus 57.3%; p = 0.013). As shown in Table 2, regression analysis using these caregiver and child characteristics revealed that a child with malaria/fever had only about half the chance of being perceived as severely or very severely ill compared to a child with other illnesses (Odds Ratio (OR) = 0.469; Confidence Interval (CI) = 0.273 - 0.805; p = 0.006). The other child and caregiver characteristics were not statistically significant.

**Table 2:** Logistic regression analysis: caregivers' opinion on the severity of child's illness

Independent Variable^1^	Illness Severe/Very Severe Number (%)	Odds Ratio	95% Confidence Interval	p-value
**Characteristics of Caregiver**				

**Age (n = 256)**				
20 years & below*	24 (61.5)	1	0.410 - 2.935	0.855
21 years & above	133 (61.3)	1.096		

**Parity (n = 234)**				
1 - 2 children*	63 (58.9)	1	0.447 - 1.659	0.656
3 children & above	77 (60.6)	0.861		

**Marital Status (n = 264)**				
All Other Statuses^2 ^*	141 (63.5)	1	0.754 - 3.790	0.202
Monogamy	22 (52.4)	1.691		

**Income (n = 253)**				
Below Ksh.5000*	108 (60.7)	1	0.325 - 1.216	0.168
Ksh.5000 & above	51 (68.0)	0.629		

**Education (n = 263)**				
Primary & Below*	142 (64.0)	1	0.905 - 4.790	0.084
Secondary & Above	20 (48.8)	2.082		

**Occupation (n = 261)**				
Unemployed*	57 (61.3)	1	0.766 - 1.508	0.676
Employed/Trader/Farmer	106 (63.1)	1.075		

**Characteristics of Child**				

**Age (n = 264)**				
6 months & below*	27 (56.3)	1	0.270 - 1.289	0.185
Above 6 months	137 (63.4)	0.590		

**Gender (n = 259)**				
Male*	73 (61.3)	1	0.660 - 2.162	0.557
Female	86 (61.4)	1.195		

**Complaint (n = 253)**				
Non-Malaria/Febrile Illness^3^*	67 (57.3)	1	0.273 - 0.805	**0.006**
Malaria/Febrile Illness	93 (68.4)	0.469		

^1^All variables entered in the first step

^2^Includes: polygamous marriage, single parent, divorced or separated and widowed

^3^Non-Malaria/Febrile Illnesses: respiratory tract infections/cough, diarrhoea, and skin diseases

*Baseline group

Caregivers were asked about what actions they took when they first noticed their child was ill. Their responses are summarized in Table 3 which shows that the most common first action was to buy drugs from shops (53.1%), while private hospitals were the least utilized (1.2%). The type of first action taken was disaggregated into two groups of those caregivers who bought drugs from the local shop/kiosk and those who took their children to any health facility. The caregiver characteristics showed no statistically significant association with actions taken.

**Table 3:** The First Action Taken for a Sick Child (n = 256)

Action	Frequency	Percent
Bought medicine from shop	136	53.1
Took child to govt. hospital	68	26.6
Took child to private hospital	3	1.2
Took child to traditional healer	16	6.3
Other actions	4	1.6
Took no action	29	11.3

**Total**	**256**	**100.0**

The data also show that 11.3% of caregivers reported not taking any action. Qualitative data revealed that caregivers sometimes failed to take their children for treatment due to reasons ranging from lack of money to finance transportation and user fees at facilities, past experiences with poor services offered and a failure to appreciate the severity of illnesses. During FGDs, caregivers reported that certain illnesses were commonly taken to traditional healers. Although the exit interviews revealed a low utilization of the traditional healers (6.3%), the impression created by FGD participants was that traditional healers were a widely consulted group of practitioners who continued to hold a place in determining the fate of certain groups of children with particular illnesses whose nature we could not verify. To illustrate this point, a few quotes from different FGDs are given below:

"One can give birth to a baby with certain teeth in the mouth. If you take to the hospital, the doctors don't know how to remove these teeth. One has to take to a traditional doctor; there is a way in which they cut them"

*"A child can be small, he can be eating but the body is just dry. There are doctors, who hold the baby in a certain way, then they pierce the body between the breast and ribs, they apply something and the body of the child returns *[improves]. *And this, if you take to the hospital, there is nothing they will do,"*

*"Ebikhokho, must also be taken to the *[traditional healer] *who cuts the baby for the child to be well. If you take the child with ebikhokho to the hospital he will die, especially if they are injected,"*

The study found that caregivers follow interventions already in place to prevent childhood illnesses such as the use of mosquito nets and immunization, as well as breastfeeding. For instance, a majority of the children (80.6%) was reported to have spent the previous night under a mosquito net. Only 19.4% reported their child had not slept under a mosquito net. Among the 77 respondents whose children had not used a mosquito-net the previous night, 32 (41.6%) reported not owning any mosquito net at home. Further analysis showed that the caregiver's age, marital status, education level and occupation and the child's age and gender were significantly associated with the child sleeping under a mosquito net. Caregivers' income level and parity did not show any statistical significance in predicting a child's mosquito net use. In order to control for confounding, stepwise logistic regression analysis was carried out and the results showed that if the caregiver was in a monogamous marriage, then their child was more than twice as likely to have slept under a net than those of other marital statuses (OR: 2.702; CI: 1.343 - 5.434; p = 0.005); and if the caregiver was employed or was a trader, then the child was almost twice as likely to have slept under a net (OR 1.851; CI 1.285 - 2.664; p < 0.001). The results also showed that the younger children (aged less than 6 months) and boys were less likely to have slept under a net than their older siblings and the girls.

In regard to immunization, a majority of the respondents (90.2%) had the "Road to Health" card in which the health workers record vaccinations administered to the child. A cross-check of these cards revealed that up to 97.8% of them were up to date with vaccinations appropriate for age. Almost all the caregivers without cards for their children said that their children's vaccination statuses were up to date.

**Figure 2:**
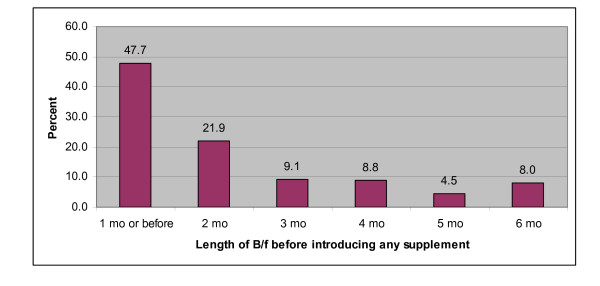
**Respondents' Opinions on Length of Exclusive Breastfeeding**.

Caregivers were also asked about their opinions on the length of exclusive breastfeeding on the mother's breast milk before a child is introduced to any other form of supplementary feeding. Their responses as summarized in Figure 2 show that 78.7% started supplementation within the first three months of life and almost a half (47.7%) started before the end of the first month. A majority of FGD discussants said it was common practice to start supplementation very early in the child's life in this area. Further probing revealed various reasons for this practice:

"It depends on the type of child. Some come out of the womb when they are very hungry and cry a lot. So these must be given food almost immediately"

A majority (98%) of exit interviewees continued breastfeeding during their child's illness, a fact that was corroborated in qualitative interviews where participants revealed that mothers knew they should continue to breastfeed if the child was ill, but the practice was dictated by the type of illness. For instance, if the child was vomiting or lost appetite or had a particular oral pathology referred to locally as "*obuyindi"*, they would usually stop breastfeeding until the child recovered.

"*We cannot force them to breastfeed when they have no appetite*"

"*Milk is not good when a child is vomiting and has diarrhoea. We normally stop and give ORS first before we continued to breastfeed*"

### Perceptions of Health Care

The single most experienced problem at the health facilities mentioned by 78.4% of all respondents was the long waiting time. Other problems mentioned included services not being up to expected standards (poor services) (5.8%), lack of drugs (9.6%) and unfriendly or rude staff (6.2%).

We further investigated which independent variables were more likely to predict whether a caretaker would report having experienced problems at a health facility (Table 4). The results revealed that caregivers' income and education level were the strongest predictors of their reporting problems experienced at health facilities. About 80.2% of those who earned 5,000 shillings and above vs. 63.2% who earned less, and 80.4% of caregivers with secondary or higher education level vs. 65.8% of those with lower education level, reported having experienced problems at the health facilities. These differences were highly statistically significant with p = 0.001 and 0.031 for income and education status, respectively. Although there was a ten percentage point difference between the two marital status groups in reporting problems experienced at health facilities (monogamous 66.0% and other marital statuses 76.1%), this difference was not statistically significant.

**Table 4:** Characteristics of those who reported experiencing problems at health facility

Variable	Number (%)	Chi Square Value	p-value
**Caregiver characteristics**			

**Age (n = 385)**			
20 years & below	42 (64.6%)	0.425	0.515
21 years & above	220 (68.8%)		

**Parity (n = 350)**			
1 - 2 children	112 (70.4%)	0.140	0.708
3 children & above	131 (68.6%)		

**Marital Status (n = 396)**			
All Other Marital Statuses	51 (76.1%)	2.6	0.105
Monogamy	217 (66.0%)		

**Income (n = 382)**			
Below Ksh.5000	168 (63.2%)	10.805	**0.001**
Ksh.5000 & above	93 (80.2%)		

**Education (n = 395)**			
Primary & Below	223 (65.8%)	4.680	**0.031**
Secondary & Above	45 (80.4%)		

**Occupation (n = 393)**			
Unemployed Employed/Trader	94 (69.6%) 105 (64.4%)		
Farmer	68 (71.6%)	1.683	0.431

**Characteristics of the child**			

**Age (n = 395)**			
6 months & below	75 (67.0%)	0.056	0.813
Above 6 months	193 (68.2%)		

**Gender (n = 391)**			
Male	121 (66.5%)	0.042	0.837
Female	141 (67.5%)		

**Complaint (n = 388)**			
None	97 (73.5%)	2.803	0.246
Non-Malaria/Febrile Illness^1^	92 (67.2%)		
Malaria/Febrile Illness	76 (63.9%)		

^1^Non-Malaria/Febrile Illnesses: respiratory tract infections/cough, diarrhoea, and skin diseases

Opinions of FGD participants on whether the health system acted as a barrier to accessing healthcare were varied, with overall consensus being on the issues of understaffing and lack of drugs as the key barriers that forced them to seek alternative treatments for example, from traditional medicine men, herbalists, and hawkers. In some of the FGDs, the caregivers narrated their experiences at the hands of health staff, whom they thought acted rudely or unprofessionally as a result of being overworked from understaffing. On the other hand, one KI interviewee said that the health system did not pose a barrier but that it was the caregivers who failed to understand the system. There was agreement also on the "good side" of the health care system, which cast a good image on preventive aspects of care, for example, immunization and family planning and a bad one for curative departments. Overall, discussants were of the opinion that the curative departments were the worst staffed hence the few members of staff could not give adequate or expected care. This was best illustrated by one discussant who said:

*"....the clinic side is ok, if you go there; you are attended to very well. On my side, there is a problem with treatment side. When you go with the child, you become the doctor. You will sit with the baby as you are asked about the baby. So you will tell him how the baby is coughing, how the temperature is high and so on, but there is no doctor *[health worker] *who will take their time to examine the baby in order to know whether it is the breast or the ribs *[that have a problem], *there is no good service here."*

Despite all the foregoing, participants on the other hand portrayed serious misconceptions about how services are or should be offered at the health facilities.

*"...But he will not examine the child. In case he does, then you know that the child is very serious and will die any time soon..... If he takes that thing *[stethoscope] *and places it on the child's chest, then that child will surely die"*.

Other notable misconception was the belief in injections as the best form of treatment, a fact that some key informants said was exploited by quacks, who treated all illnesses with injections. Additionally, the key informants said it was ignorance of the caregivers who failed to understand standard treatment protocols, resulting in unnecessary complaints. But the misconceptions were coupled with what caregivers viewed as incompetence of either health workers or the system:

*".... but you yourself will have to be the doctor. You tell him how the child is and his work is to write in the book. Then he says: bring needle, bring syringe*, [now] *go for injection. But when it comes to the injection, maybe there is no drug, you are asked to go and buy. Now in this case, are you the one helping him or he is the one supposed to be helping you?"*

Rudeness and harshness on the part of the health worker was also mentioned as a potential inhibitor of the caregivers wanting to continue to use the health facilities for care of their children. For instance in some FGDs, the caregivers had the following to say:

*"At *[our facility], *just like one member has said, you can arrive with a child and then one nurse starts to scold you: 'Where have you been with this child?' You can also get annoyed because if you were not recognizing their services, you wouldn't have brought the child."*

*"You can go and find one who is very harsh and however sick your child is, you will just want to turn back and go home with them, saying 'Aah, if it is *[this one] *here today, let me go back home, I will only come back when somebody else is on duty'."*

One of the KI questions was to find out whether the health system was acting as a barrier to accessing healthcare. While it was clear from discussions that the long waiting time, staff shortage, lack of drugs and unfriendly staff were some of the widespread occurrences within the study sites, suggestions on how to improve and manage certain aspects of the health care delivery system varied among the different groups of respondents, with those of exit interviewees being summarized in Table 5. While about 5.3% of them specifically suggested supervision of staff, virtually all the KI interviewees were of the opinion that facility-level supervision of the staff to improve their performance and output would not work well.

**Table 5:** Respondents' Opinions on How to Improve Access to Healthcare for Under-fives†

Recommendation	Count†	Percent
Increase staff	267	41.5
Expand facility & space	125	19.4
Avail drugs	129	20.1
Increase opening hours to 24 hrs	45	6.9
Train staff to be caring; Increase supervision	34	5.3
Install electricity, Provide water	29	4.5
Proper triage	6	1.0
Educate mothers on health care	3	0.5
Provide free nets	3	0.5
Improve roads	2	0.3

**Total**	**643**	**100.0**

†Multiple response question

*"... also, it [supervision] won't work. Somebody like me, how can you supervise me? That one can't work. I will not do a good work when supervised. That one, I don't want to cheat you..... There are things that have been supervised somewhere *[else] *and they failed."*

## Discussion

### Study Limitations

Conducting exit-interviews might have introduced a bias in presenting the views of only those that overcame certain barriers. However exit interviews were complemented by FGDs held in the community, and most results from the exit interviews concurred with issues raised in FGDs. Selection bias was therefore minimized. A community survey in the study area would have been more appropriate, but was not done due to time and financial constraints.

Some questions required a re-call time on the part of caregivers and answers to these might have been influenced by re-call bias. To minimize this, the interviewers were carefully trained to ask the questions exactly as written and to probe or provide explanations whenever it seemed that the interviewee did not understand.

### Socio-demographic Findings

The main socio-demographic findings revealed a high proportion of female caregivers, which is not surprising in this setup as tradition has often bestowed the responsibility of child-caring on women, especially in the rural areas like Butere. The low educational attainment and high rate of unemployment suggest a low socio-economic status of the rural residents.

The youngest mother interviewed was reportedly 10 years and at least 13 respondents (mothers) were aged below 18 years. Under age mothers were not excluded from the study since we sought to determine those maternal characteristics important to children's survival. Capturing under-age mothers at the point of exit is in itself important data showing that first, childbearing starts early and second, children take care of other children in these settings, facts which in combination lead to high childhood morbidity and mortality.

Conversely, the fact that under-age mothers were interviewed might in itself raise ethical issues on their ability to give valid consent for interview. However, the fact that underage mothers take care of children can be argued to be raising both social capital and economic challenges for them to access healthcare, and so it was very important to get their side of the story. Additionally, these mothers are considered to be biologically underage, but socially, they are adults in the sense that the society has bestowed upon them the responsibility of bearing children and taking care of them. Whether they take on this responsibility successfully is the core issue of this inquiry. In this community, and indeed in many other African settings, one is considered an adult once they start bearing children, regardless of their biological age [20].

Kenya has been implementing youth-friendly services in some hospitals but this has been on a small scale, only targeting services related to HIV/AIDS and sexually transmitted infections. If these services were upscaled to primary-level facilities and incorporate a wider range of services including ANC, child welfare and family planning services, then the youth, who are potential young mothers could also access them, thereby reducing potential early motherhood and the related negative consequences.

### Perceptions and Practices of Caregivers Concerning Childhood Illnesses

The ability of the caregiver to recognize a sick child has been associated with good response in terms of appropriate actions taken [21,22]. The first actions taken by caregivers in response to children's illnesses in this study were typical of other findings [21,23-27], suggesting that home-management of childhood diseases with shop-bought medicines is a common practice for most rural populations.

Exit interview data showed that a small proportion of caregivers sought the services of traditional healers, which corroborates results of an earlier survey done in Western Kenya [27]; but qualitative data suggest a different pattern. Specific childhood diseases such as *ebikhokho, kilimi *and *obuyindi *(whose nature we could not determine as even the health workers had differing views on what these really were) were commonly treated by traditional healers. Additionally, some health workers used to refer caregivers to the traditional healers whenever they suspected a child to be suffering from any of the listed illnesses. As such, the rate of utilization of the traditional healers is likely to be higher among the general population than that reported in this study. There is need for more research on why caregivers prefer traditional healers for certain childhood illnesses, and these might provide valuable lessons regarding the appropriate handling of mothers and children in this setting, and indeed in many other rural areas of Africa.

The rate of mosquito-net usage is high compared to other studies done in Kenyan rural areas [11,28] where the rates were between 15% and 33%. However, the factors predicting whether a child had slept under a mosquito net were similar. This high proportion may be a reflection of the true situation since the social marketing strategy by Population Services International (PSI) adopted nationally in 2001 may have achieved its program target [29], as indicated in Noor *et al *[11]. On the other hand, the self-selected sample of caregivers attending the health facilities may have been biased towards caregivers exposed to these interventions. However, this type of sample has been used previously with different results, although the timing was prior to mass distribution of ITNs [28]. Nationally, the country seems to be moving in the right direction, with under-five mortality declines being attributed partly to increased net coverage. The most recent demographic and health survey indicates that indeed, there have been improvements in ownership and use of ITNs countrywide with over half of children under age five sleeping under a net the night prior to the survey in 2008-09 compared with only 15 percent in 2003 [5]. It is therefore possible that mosquito net coverage within our study population is close to that indicated.

It was interesting to note caregivers' opinions on how long a child should be exclusively breastfed. More than 90% of caregivers felt it was appropriate to introduce supplementation earlier than recommended. Literature is replete with evidence of the advantages of exclusive breastfeeding for a period of at least 6 months, among them less morbidity from gastrointestinal infections and mothers experiencing prolonged lactational amenorrhea which is important in child spacing and better survival [30]. It is obvious that this community may never experience these benefits if the reported practice continues.

### Health System Barriers to Healthcare

Among the barriers related to the health care delivery system mentioned explicitly by caregivers in exit interviews were long waiting time, lack of drugs and poor services, among others. During FGDs, additional barriers mentioned included incompetence and perceived poor attitude of the health workers. Conversely, FGDs also elicited widespread misconceptions among the caregivers about healthcare provision; these were confirmed by key informants reporting a widespread ignorance among the community members.

Experience of poor services was often viewed by caregivers as a constraint to seeking health services. Sobo et al, observed that the outcomes of experiencing access barriers contributed to suboptimal utilization of the health care system by the caregivers [31], an observation that has been confirmed by this study. Therefore, in the case where caregivers are not fully involved in the care of their children and are treated as illiterate observers or the health workers are rude or harsh, then access to healthcare for caregivers' children is greatly compromised. In other settings, some parents have had to deal in their own ways with what they saw as inexcusable incompetence among health workers [31]:

"I am expecting that the doctor is going to check the boy's ears, his temperature, I don't know - he doesn't do anything. He looks in, and asks how he is doing and I say 'The same', then he prescribes another thing ... or they go to their books and start reading, to see what they are going to prescribe, what does he have - if I had a book on my side, then I wouldn't need to come to the doctor."

## Conclusions

This study aimed to investigate and document the barriers to access to healthcare services for children aged less than five years in Butere. The results complement similar studies done elsewhere which show that certain caregiver-related attributes determine early childhood illness recognition and the taking of appropriate actions. New evidence from this study shows that the healthcare delivery system poses recurrent barriers to the accessing of healthcare for the under-fives, while caregivers expect more than what is offered in terms of quality of care. On the other hand, caregivers exhibited multiple misconceptions about the care that is offered at public health facilities.

For these reasons we would like to recommend firstly that the caregivers need to be empowered by the system comprising of local care managers in order to not only recognize but also to respond appropriately to illnesses in young children. Secondly, the MOH can deal with perennial staff shortages through, for example, incorporating the alternative health-care delivery practitioners (medicine men, quacks and TBAs among others) into the mainstream health-care, through training and systematic registration and recognition in collaboration with the Ministry of Social Services. At the national level, the government can implement a rural health facility-specific incentive to help retain qualified health professionals at the rural facilities, and this would improve service delivery at the primary health facilities.

Thirdly, the MOH, district health managers, the Kenya Medical Supplies Agency (KEMSA) and other relevant authorities can combine efforts in ensuring that the quality of service delivery is up to at least the minimum expected standards. The MOH can ensure this through regular sharing of information on current practices; the district health managers can ensure that facilitative supervision and support takes place in their areas of jurisdiction; and KEMSA could improve its supply chain management and delivery of drugs and other medical supplies to health facilities.

If these recommendations are implemented, we are likely to see a shift towards positive gains in the delivery of healthcare to the under-fives in the country, hence tackle the high mortality and pace towards achieving the fourth MDG.

## Competing interests

The authors declare that they have no competing interests.

## Authors' contributions

ASO conceived and designed the study, participated in data collection and analysis, and wrote the first draft of the manuscript and contributed to its revision. LON, JMO and AMRL contributed to data analysis, writing and revising the final manuscript. All authors read and approved the final manuscript.

## Pre-publication history

The pre-publication history for this paper can be accessed here:

http://www.biomedcentral.com/1471-2458/11/272/prepub
